# Longitudinal analysis of surgical outcome in subjects with pulsatile tinnitus originating from the sigmoid sinus

**DOI:** 10.1038/s41598-020-75348-3

**Published:** 2020-10-23

**Authors:** Sang-Yeon Lee, Min-Kyung Kim, Yun Jung Bae, Gwang Seok An, Kyogu Lee, Byung Yoon Choi, Ja-Won Koo, Jae-Jin Song

**Affiliations:** 1 Department of Otorhinolaryngology-Head and Neck Surgery, Seoul National University Bundang Hospital, 300 Gumi-dong, Bundang-gu, Seongnam, Gyeonggi-do 13620 Republic of Korea; 2 Department of Radiology, Seoul National University Bundang Hospital, Seongnam, Republic of Korea; 3 Music and Audio Research Group, Graduate School of Convergence Science and Technology, Seoul National University, Seoul, Republic of Korea

**Keywords:** Medical research, Neurology, Signs and symptoms

## Abstract

A dominant sigmoid sinus with either diverticulum or dehiscence (SS-Div/SS-Deh) is a common cause of pulsatile tinnitus (PT). For PT originating from SS-Div/SS-Deh, an etiology-specific and secure reconstruction using firm materials is vital for optimal outcomes. As a follow-up to our previous reports on transmastoid SS resurfacing or reshaping for SS-Div/SS-Deh, this study aimed to evaluate the long-term results of transmastoid resurfacing/reshaping. We retrospectively reviewed 20 PT patients who were diagnosed with SS-Div/SS-Deh, underwent transmastoid resurfacing/reshaping, and were followed up for more than 1 year postoperatively. For PT, immediate and long-term changes (> 1 year) in loudness and annoyance were analyzed using the visual analog scale (VAS). Additionally, pre and postoperative objective measurements of PT using transcanal sound recording and spectro-temporal analysis (TSR-STA), imaging results, and audiological findings were comprehensively analyzed. Significant improvements in PT were sustained or enhanced for > 1 year (median follow-up period: 37 months, range: 12–54 months). On TSR-STA, both peak and root mean square amplitudes decreased after surgery. Also, the average pure-tone threshold at 250 Hz improved after surgery. Thus, our long-term follow-up data confirmed that the surgical management of PT originating from SS-Div/SS-Deh is successful with regard to both objective and subjective measures.

## Introduction

Tinnitus, characterized by a conscious auditory perception in the absence of an external stimulus, is a common otological condition^1^. It can be classified as pulsatile tinnitus (PT) and non-pulsatile tinnitus, according to the perceived sound quality^2^. While non-pulsatile tinnitus is thought to be a consequence of functional changes in the auditory and the non-auditory cortices^3,4^, PT is due to major vascular wall anomalies in the temporal bones^5,6^. Vascular PT is characterized by an auditory perception of pulse-synchronous sound. There are three potential mechanisms by which vascular wall anomalies may cause PT: (1) an alteration in the vascular hemodynamics may cause turbulent blood flow and lead to sound transmission^7,8^; (2) vibration of a dehiscent vascular wall even in the absence of turbulent flow may occur^9,10^; (3) abnormal sound perception may occur due to third-window lesions in the inner ear^11,12^. Therefore, the identification of the causative vascular pathology and etiology-specific management are vital for the optimal treatment of PT patients.

Sigmoid sinus dehiscence and/or diverticulum (SS-Div/SS-Deh), one of the most common identifiable vascular abnormalities causing vascular PT, occurs in approximately 18% of PT patients^13,14^. Surgical procedures for transmastoid sigmoid sinus (SS) resurfacing/reshaping employ a multistep reconstructive process, including external decompression or reduction of the SS, reconstruction of a sound-proof barrier using a firm material, and disconnection of the sound transmission^5,15^. Although preliminary studies reported successful treatment outcomes^5,15^, the lack of longitudinal follow-up studies and an objective assessment of treatment outcome leaves the long-term efficacy of the surgical treatment unclear. Moreover, upstream venous stenosis has recently been presumed to be associated with PT perception in patients with SS-Div/SS-Deh^13^. This, in turn, implies that a reemergence of symptoms that initially subside after surgery may be observed because upstream stenosis-induced high jet flow may result in an increased irregularity or asymmetry in the morphology of the SS^16^.

As a follow-up study to our aforementioned initial studies, we herein evaluate the long-term efficacy of SS resurfacing/reshaping techniques in a relatively large cohort with SS-Div/SS-Deh. By reviewing the results of objective assessments, including transcanal sound recording with spectro-temporal analysis (TSR-STA) and pure-tone audiometry (PTA), and correlating these with subjective symptom improvements, we suggest possible pathophysiological mechanisms of PT perception and its long-term improvement after surgical managements. Our results provide a novel insight into the effect of stringent selection of surgical candidates among PT patients with SS-Div/SS-Deh and patient-tailored surgical approaches on successful treatment.

## Results

### Demographic and clinical characteristics

**Figure 1:**
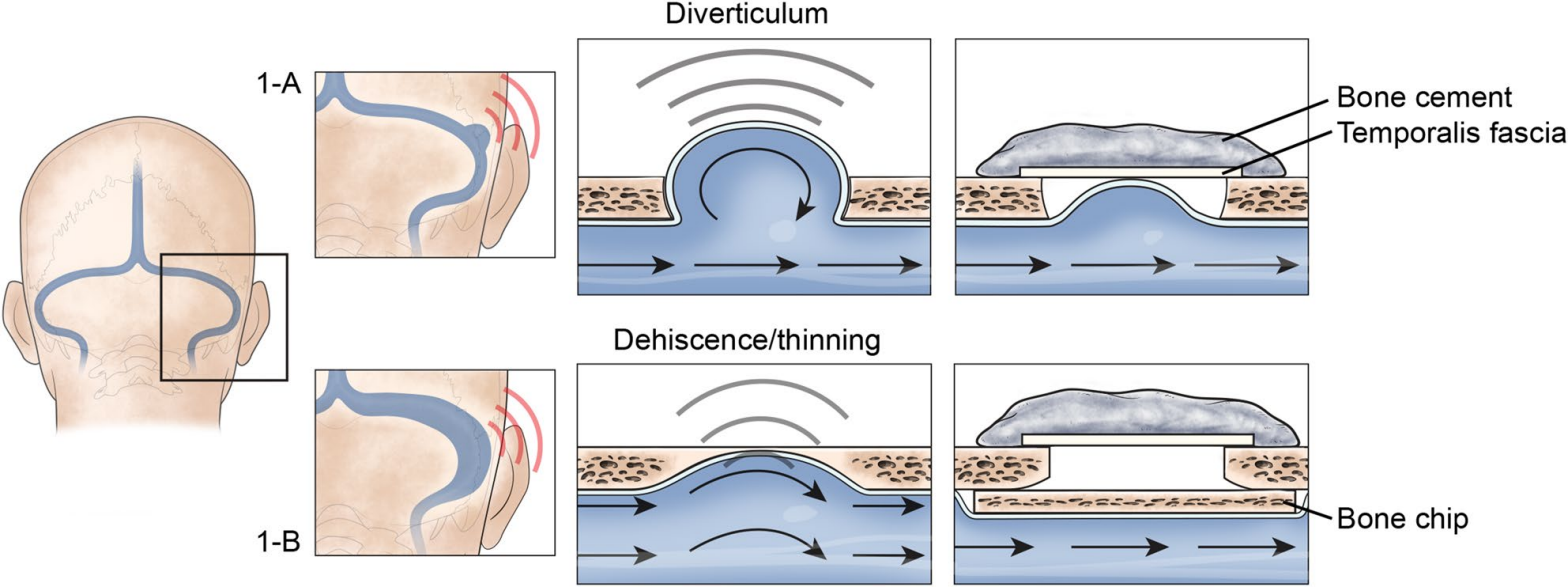
(**a**) Schematic illustration of the surgical procedures of the transmastoid sigmoid sinus (SS) resurfacing surgery for patients with SS diverticulum (SS-Div). (**b**) Schematic illustration of the surgical procedures of transmastoid SS reshaping for patients with SS dehiscence (SS-Deh). A multistep reconstructive process, including external reduction (**a**) or compression (**b**) of the sigmoid sinus, reconstruction of a sound-proof barrier using a firm material (i.e., bone cement), and disconnection of the sound transmission, may be the keys to quieting PT.

A schematic illustration of the transmastoid SS resurfacing for SS-Div and reshaping for SS-Deh techniques that we have previously reported are summarized in Fig. 1. The clinical characteristics of the 20 patients enrolled in this study are summarized in Table 1. The mean age was 42.9 ± 13.0 years (range: 22–73 years), and 18 of 20 subjects were female. The mean body mass index (BMI) was 23.9 ± 3.7 (range: 18.17–33.97). Sixteen of twenty patients presented with right-sided PT. The mean duration of PT was 25.0 ± 35.4 months (range: 2–135 months). TB-CTA confirmed definite SS-Div/SS-Deh localized to the affected side of PT. According to TB-CTA, 13 (65%), 6 (30%), and 1 (5%) patients showed SS-Deh, SS-Div, and combined SS-Div/SS-Deh, respectively. Intraoperatively, 11 of the 14 subjects with SS-Deh exhibited true bony dehiscence at the junction between the transverse sinus and the SS, whereas the other three showed focal thinning of the SS wall without any dehiscence. Of the 15 patients who underwent brain magnetic resonance imaging/angiography (MRI/MRA), 4 (26.7%) had transverse sinus stenosis (TSS) and 1 (0.7%) had Empty Sella (Fig. S1).

**Table 1:** Demographics and clinical characteristics.

Subject	Age/Sex	PT duration	BMI	Side	Final diagnosis	Operation	Radiologic test		Objective tests			Subjective tests					
							Empty sella	TSS	Pre-pseudo LFH at 250 Hz	Post-pseudo LFHL at 250 Hz	TSR/STA	Preop		Short		Long	
												VAS Loud	VAS Annoy	VAS Loud	VAS Annoy	VAS Loud	VAS Annoy
1	55/F	2	24.62	R	SS-Deh	SS reshaping	NA	NA	(−)	(−)	NA	5	5	0	0	0	0
2	33/F	13	22.24	L	SS-Deh	SS reshaping	NA	NA	(−)	(−)	NA	10	10	8	8	8	8
3	36/F	6	18.17	L	SS-Div	SS resurfacing	NA	NA	(−)	(−)	NA	6	7	3	3	3	3
4	62/M	68	30.59	R	SS-Div	SS resurfacing	Partial	(−)	(−)	(−)	NA	9	9	10	6	4	4
5	41/F	3	25.83	R	SS-Div	SS resurfacing	(-)	(−)	(−)	(−)	NA	3	2	0	0	0	0
6	41/M	4	26.33	L	SS-Deh Dural AVF^a^	SS reshaping	Partial	(−)	(−)	(−)	NA	6	6	6	3	6	3
7	39/F	4	23.88	R	SS-Deh	SS reshaping	(−)	(−)	(−)	(−)	(+)	1	2	1	2	0	0
8	42/F	74	20.43	R	SS-Div	SS resurfacing	Partial	(−)	(+), 55 dB HL	(+), 5 dB HL	(+)	10	10	1	0	0	0
9	54/F	36	22.92	R	SS-Div	SS resurfacing	Partial	(−)	(−)	(−)	NA	3	3	3	3	0	0
10	22/F	13	25.16	R	SS-Deh	SS reshaping	Partial	(+)	(+), 15 dB HL	(+), 5 dB HL	(+)	6	7	9	2	0	0
11	39/F	2	20.03	R	SS-Deh	SS reshaping	(−)	(−)	(−)	(−)	NA	8	7	8	8	4	3
12	25/F	10	21.65	R	SS-Deh HJBD^a^	SS reshaping JB resurfacing	NA	NA	(+), 25 dB HL	(+), 10 dB HL	(+)	7	7	8	8	6	5
13	35/F	4	20.28	R	SS-Deh	SS reshaping	Partial	(−)	(−)	(−)	(+)	5	5	2	2	0	1
14	28/F	13	25.49	R	SS-Div	SS resurfacing	Partial	(+)	(−)	(−)	NA	7	7	3	2	2	1
15	48/F	74	25.86	R	SS-Deh	SS reshaping	Partial	(−)	(+), 20 dB HL	(+), 5 dB HL	NA	7	7	5	5	3	2
16	56/F	8	20.73	R	SS-Deh	SS reshaping	(−)	(−)	(−)	(−)	NA	4	4	7	7	3	3
17	32/F	5	22.9	R	SS-Deh	SS reshaping	Empty	(+)	(+), 40 dB HL	(+), 15 dB HL	NA	8	8	2	1	1	1
18	32/F	135	33.97	L	SS-Deh	SS reshaping	Partial	(−)	(−)	(−)	NA	7	8	1	2	1	1
19	45/F	13	24.65	R	SS-Deh	SS reshaping	Partial	(+)	(+), 25 dB HL	(+), 15 dB HL	NA	8	7	0	0	0	0
20	73/F	12	22.12	R	SS-Deh	SS reshaping	NA	NA	(−)	(−)	NA	7	7	2	2	2	2

Abbreviation: PT, pulsatile tinnitus; BMI, body mass index; TSS, transverse sinus stenosis; LFHL, low-frequency hearing loss; TSR/STA, spectro-temporal analysis using short-time Fourier transform; VAS, visual analogue scale; Loud, loudness; Annoy, annoyance; M. male; F, female; R, right; L, left; SS, sigmoid sinus; AVF, arteriovenous fistula; HJBD, high jugular bulb dehiscence; NA, not available.

^a^Note that two subjects who manifest “slight improvement” had other vascular anomalies that may precipitate PT.

### Subjective outcomes

**Figure 2:**
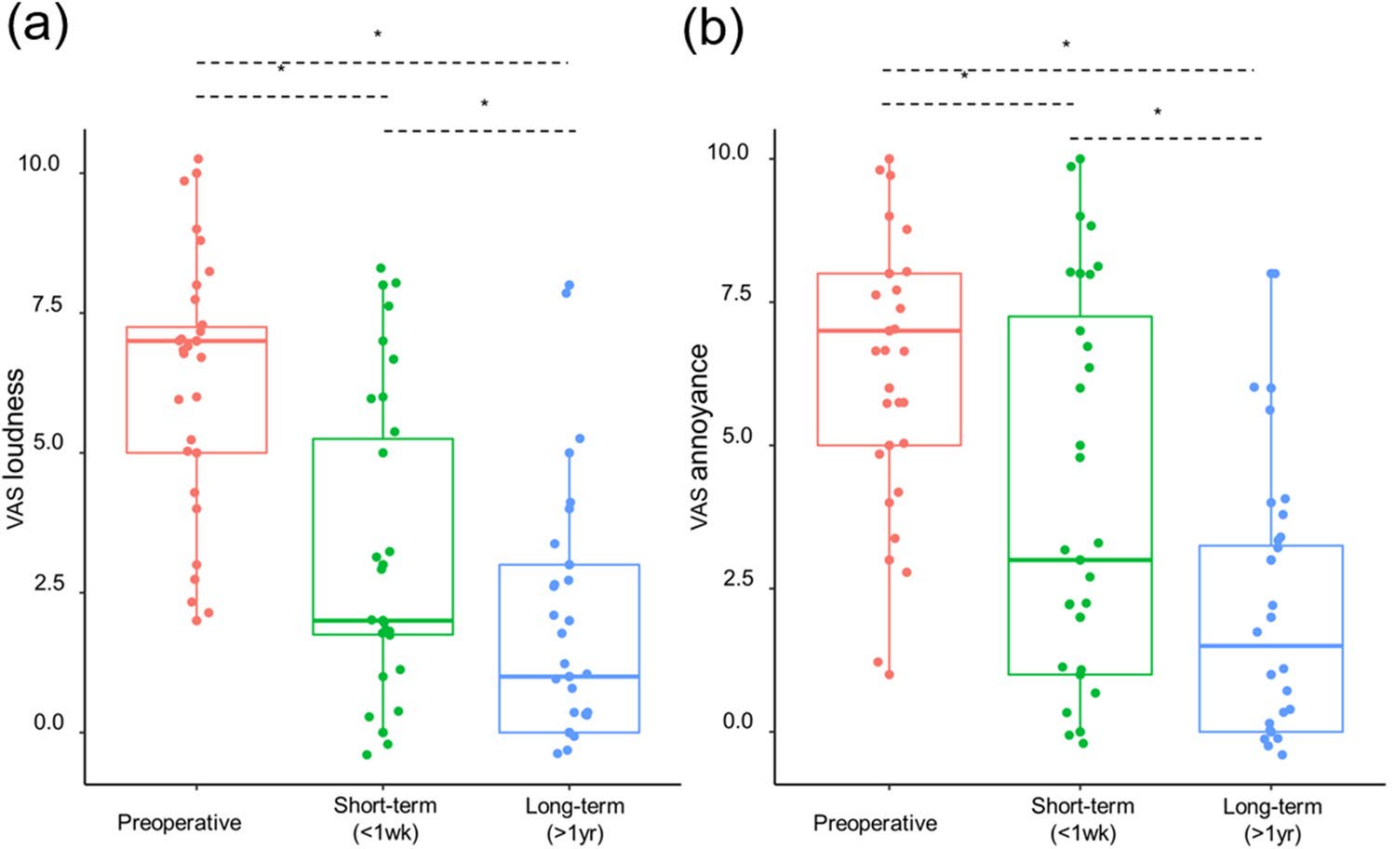
The short-term (< 1 week) and long-term (> 1 year) changes in the Visual Analog Scale (VAS) of tinnitus loudness and tinnitus-related distress are depicted. The mean VAS loudness (**a**) and VAS annoyance (**b**) significantly decreased immediately (within 1 week of surgery) and then improved over 1 year. Compared with the short-term time point (< 1 week), mean VAS loudness (**a**) and VAS annoyance (**b**) were markedly decreased after surgery in the long-term (> 1 year). *Indicates statistical significance by the Paired t-test.

In this study, after SS resurfacing/reshaping, short-term (< 1 week) and long-term (> 1 year) changes in subjective symptoms were analyzed in all patients. As summarized in Fig. 2, median Visual Analog Scales (VAS) loudness decreased significantly from 7 (range: 2–10) to 2 (range 0–8) (*P* < 0.001 by Wilcoxon-signed rank test), and VAS annoyance from 7 (range: 1–10) to 3 (range 0–10) (*P* < 0.001 by Wilcoxon-signed rank test) immediately after surgery (< 1 week).Longitudinal improvements in PT judged either by median VAS loudness from 2 (range: 0–8) to 1 (range: 0–8) (*P* = 0.001 by Wilcoxon-signed rank test) or by VAS annoyance from 3 (range 0–10) to 1.5 (range: 0–8) indicated substantial improvement 1 year postoperatively (*P* < 0.001 by Wilcoxon-signed rank test), as compared with 1 week postoperatively. The median follow-up duration was 37 months, ranging from 12 to 54 months.

Long-term postoperative outcomes were determined using VAS loudness at the last follow-up (> 1 year) and were classified into four groups: “cured” (100% resolution of PT), “much improved” (50–100% resolution of PT), “slightly improved” (0–50% resolution of PT), and “stationary” (no change or aggravation). With regard to the long-term follow-up results of VAS loudness (Fig. S2), out of 20 patients, 7 (35%) and 13 (65%) showed complete and partial improvements, respectively. Among the 13 patients who showed partial improvement, 9 (45%) were sub-classified into the “much improved” group, and 4 (20%) into the “slightly improved” group. No patients exhibited aggravation of their PT during the follow-up periods. When we compared these subgroups according to long-term post-treatment outcomes (cured vs. much or slightly improved), multiple logistic regression analysis failed to show any particular factors as independently affecting improvement in either VAS loudness or annoyance (Table 2). Interestingly, 2 of the 4 patients showing “slight improvement” also showed other vascular anomalies that may precipitate PT. One of them (Subject 6) exhibited a left SS-Deh and ipsilesional dural arteriovenous fistula, and the other (Subject 12) had a right SS-Deh and ipsilesional high jugular bulb with dehiscence (HJBD). After initial SS resurfacing, Subject 12 further underwent jugular bulb resurfacing with bone cement, and finally showed a 90% improvement in the loudness of PT.

**Table 2:** Factors associated with long-term postoperative improvement (cured vs. much- or slightly improved) using multiple logistic regression analysis.

	Estimate	Standardized	Standard error	t-value	*P* value
Age	0.001	0.034	0.007	0.142	0.888
Sex	0.236	0.250	0.215	1.095	0.288
PT duration	− 0.003	− 0.235	0.003	− 1.026	0.319
BMI	− 0.018	− 0.160	0.026	− 0.687	0.501
Laterality	0.375	0.375	0.219	1.716	0.103
Presence of Empty sella	− 0.143	− 0.105	0.376	− 0.380	0.710
Presence of TSS	− 0.182	− 0.237	0.207	− 0.878	0.396
Presence of pseudo-LFHL	− 0.088	− 0.105	0.197	− 0.447	0.660

Abbreviation: PT, pulsatile tinnitus; BMI, body mass index; TSS, transverse sinus stenosis; LFHL, low-frequency hearing loss.

### Objective outcomes

**Figure 3:**
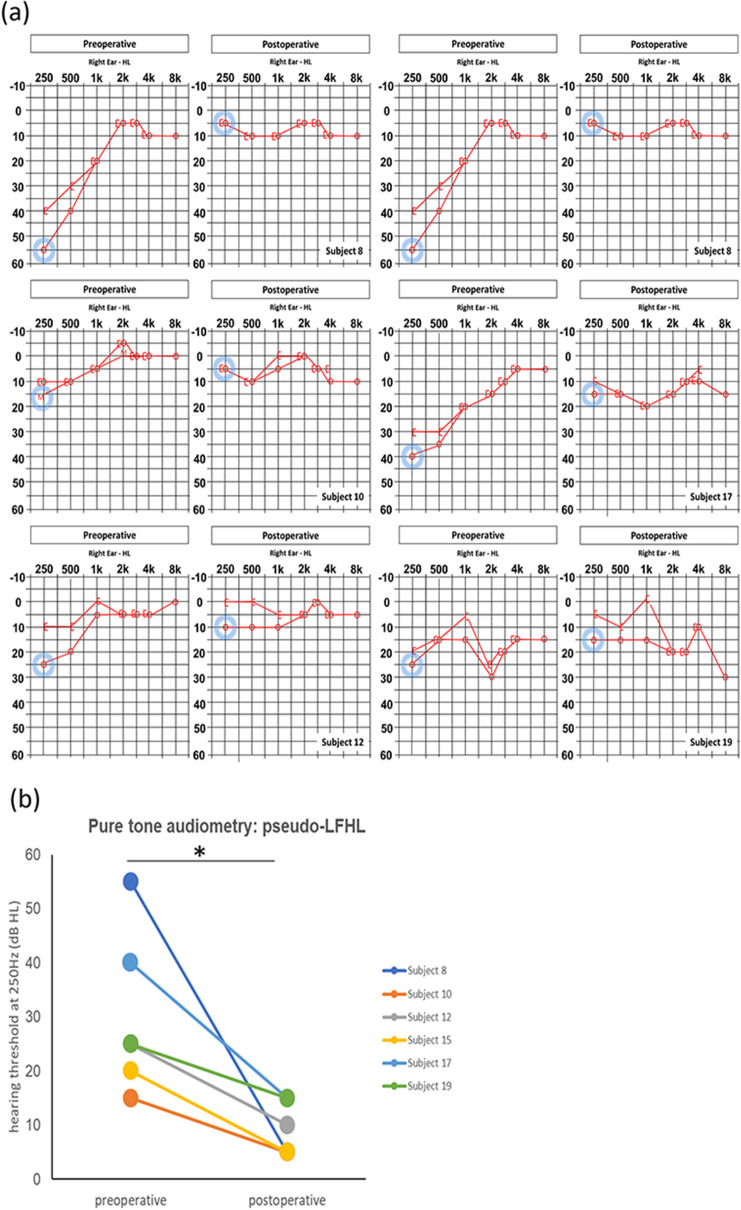
(**a**) Comparison of pre and postoperative pure tone audiometries. Of 20 patients, 6 (30%) exhibited ipsilateral pseudo-low frequency hearing loss (LFHL), as proposed by our criteria, at preoperative evaluation. Improvements in the low-frequency hearing thresholds were evident postoperatively in all 6 subjects. (**b**) Changes in hearing threshold at 250 Hz of 6 subjects with ipsilateral pseudo-LFHL. All subjects showed significant improvement in air-conduction hearing threshold at 250 Hz immediately after the operation. The blue circle indicates the LFHL at 250 Hz. *Indicates statistical significance by the Paired t-test.

All patients underwent pre and post-treatment PTA. According to our previous report^17^, pseudo-low frequency hearing loss (pseudo-LFHL) (an ipsilateral hearing threshold greater than 10 dB HL at both 250 and 500 Hz, or greater than 20 dB HL at either 250 or 500 Hz compared with the contralateral side) in the ipsilesional ear and its improvement after surgery were evaluated. Among the 20 patients enrolled, 6 (30%) with right-sided PT demonstrated ipsilateral pseudo-LFHL preoperatively (Fig. 3a). In these 6 patients, the post-treatment average threshold at 250 Hz showed a statistically significant improvement compared to the pretreatment ipsilateral average pure-tone threshold at the same frequency (mean of difference: 20.83 dB HL, t = 3.34, *P* = 0.021; paired t-test) (Fig. 3b). The other 14 patients showed no changes in pre and postoperative PTAs.

**Figure 4:**
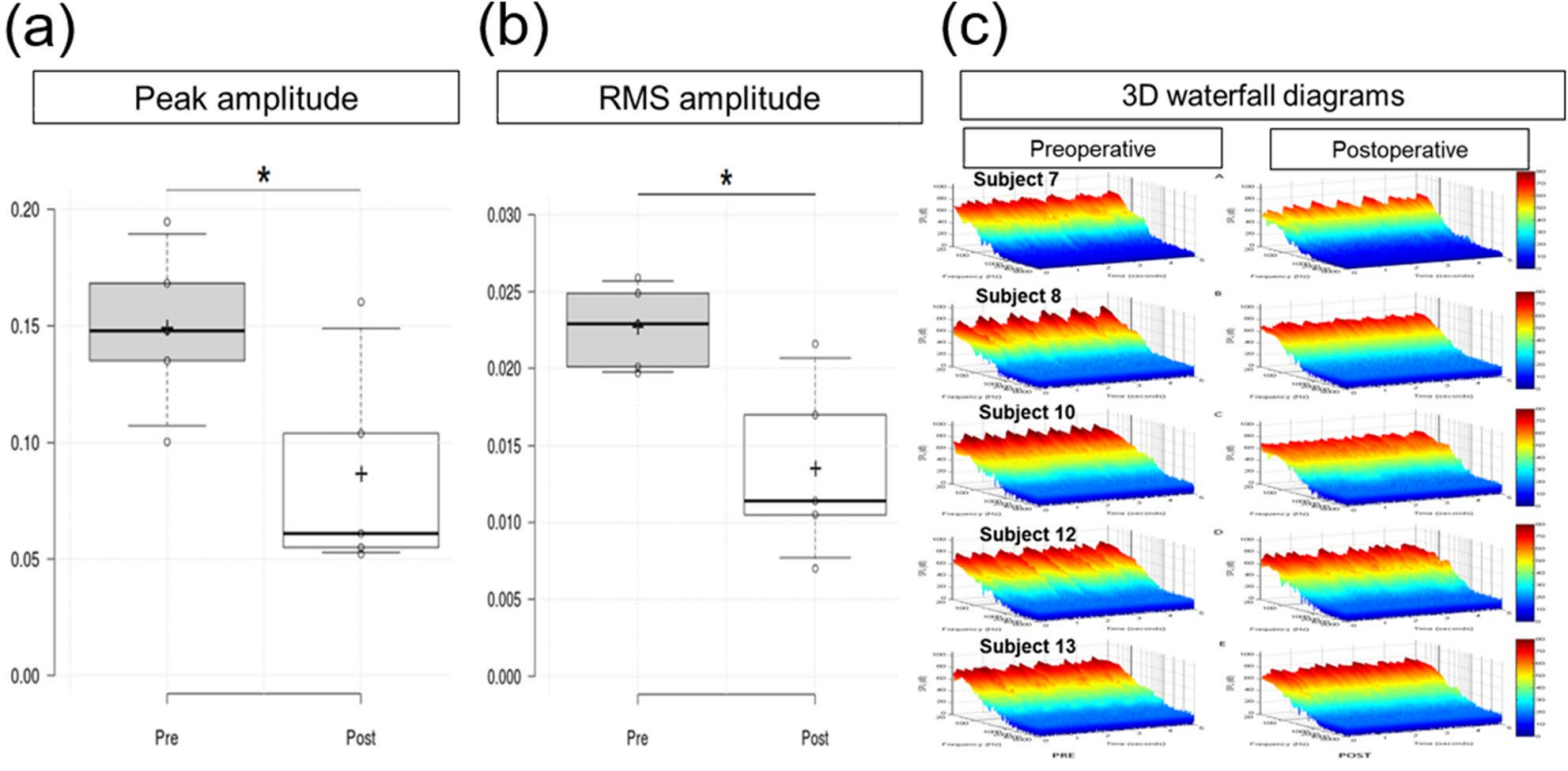
Spectro-temporal analysis of signals obtained via transcanal sound recording. (**a**, **b**) Comparison of pre and postoperative peak and RMS amplitudes. (**c**) Three-dimensional waterfall spectrograms of the pre and post-treatment signals. *Indicates statistical significance by the Wilcoxon signed-rank sum test.

Temporal analysis and spectro-temporal analysis using short-time Fourier transform (TSR-STA) was performed to objectively confirm PT, and to analyze the psychoacoustic characteristics of the PT, following published protocols^5,18^. Of the 20 patients, 5 underwent both pre and postoperative TSR-STAs. As illustrated in Fig. 4, both peak and RMS amplitudes showed significant improvement after surgical intervention, as compared with the preoperative status (Fig. 4a and b, *P* < 0.05; Wilcoxon signed-rank sum test). In addition, the 2D and 3D waterfall spectrograms of the pre-treatment and post-treatment signals in these 5 subjects are shown in Fig. 4c. The pretreatment 2D and 3D spectrograms of the 5 subjects with SS-Div/SS-Deh (Subject 7, 8, 10, 11, and 12) exhibited a pulse-synchronous broadband nature of audible SPLs (Fig. 4c, left panels). Following surgery, these pulse-synchronous signals nearly disappeared or were markedly abated in all patients (Fig. 4c, right panels).

### Complications

Immediate postoperative complications were not observed. Other than persistent residual PT, long-term morbidity or mortality was not reported during the median 37 months follow-up period (range: 12–54 months) after transmastoid SS resurfacing/reshaping surgery.

## Discussion

As a follow-up study of our own preliminary reports^5,15^, the current study has explored the long-term effectiveness of transmastoid SS resurfacing/reshaping using bone cement in patients with PT caused by SS-Div/SS-Deh. Sustained improvements up to at least 12 months with regard to PT perception and PT-related distress have been demonstrated subjectively by VAS loudness and annoyance, and objectively by changes in TSR-STA and PTA. Our results suggest that transmastoid SS resurfacing/reshaping using bone cement is a reliable surgical option in patients with SS-Div/SS-Deh, ensuring long-term resolution of symptoms without any pronounced complications.

### Possible mechanisms of PT development in patients with SS-Div/SS-Deh

Although the exact mechanism of PT development in patients with SS-Div/SS-Deh remains unclear, hemodynamic changes in the SS have continuously been implicated^19,20^. In line with the current case series, PT originating from morphologically abnormal SS on the right side is common, since the right venous system generally develops as dominant^21^. According to Poiseuille’s law^22^, an increased volumetric flow rate, indirectly proportional to the fourth power of the radius of an enlarged venous system, may increase the likelihood of turbulent blood flow in the SS. In addition, an upstream stenosis has also been suggested to be associated with PT perception in patients with SS-Div/SS-Deh^13^, as the upstream stenosis-induced high jet flow may contribute to an increased irregularity of, or asymmetry in, SS vessel diameter. This assumption has been supported by a recent study showing either a higher prevalence of TSS in patients with SS-Div/SS-Deh than that in normal controls, or superior surgical outcomes of SS resurfacing surgery in the presence of TSS^9^. In the current study, the presence of TSS was confirmed in 4 out of 15 patients, which is comparable to that in previous literature. Based on this evidence, mechanical perturbations of flow proximal to the transverse sinus are presumed to be a causal mechanism underlying PT generation^13,20^.

Subsequently, hemodynamic change-induced turbulent flow may elicit the disruption of local laminar flow and continuous outward pressure, leading to focal dehiscence over the SS bony wall (SS-Deh). This may also result in the formation or enlargement of SS-Div^9,23^. Furthermore, a recent computational modeling study has supported this mechanism by demonstrating strong components of rotational blood flow in the venous outflow tract in SSD^16^. The propagation of acoustic energy from turbulent blood flow to the cochlea and tympanic membrane can lead to PT perception in patients with SS-Div/SS-Deh. Our proposed mechanism is strongly supported by evidence showing that transmastoid SS reshaping/resurfacing surgery results in resolution or considerable improvement in most patients.

Previous researchers have also suggested a close relationship between idiopathic intracranial hypertension (IIH) and SS-Div/SS-Deh in patients with PT^24,25^. Also, PT arising from SS-Div/SS-Deh is common in women with relatively high BMI. However, the interrelationships between SS-Div/SS-Deh, PT, and IIH remain unclear. Although radiological signs associated with IIH, such as TSS and empty sella, are often observed, neuroimaging findings are not essential for its diagnosis^26^. In this study, patients who exhibited TSS or empty sella did not manifest IIH-related clinical features, suggesting a reduced likelihood of an involvement of IIH in the development of PT in SS-Div/SS-Deh.

### A sustained improvement of PT more than 1 year after transmastoid SS resurfacing/reshaping

Based on the underlying mechanism of PT perception in SS-Div/SS-Deh, transmastoid SS resurfacing/reshaping aims to treat PT by restoring laminar flow, reconstructing a sound-proof barrier, and disconnecting the sound transmission route from the SS to the middle ear. In a recent systematic review, a promising outcome of transmastoid SS resurfacing has been reported in patients with SS-Div/SS-Deh, with complete or partial improvement noted in approximately 85% of cases^20^. However, its long-term efficacy remains unclear due to a paucity of long-term follow-up studies.

Our results prove the long-term efficacy of transmastoid SS resurfacing/reshaping as a substantial decrease in PT was observed immediately after surgery, and this improvement was sustained, or even progressed, for at least 1 year. Furthermore, no patients exhibited changes or aggravation of their PT during the long-term follow-up period. Notably, our long-term treatment outcomes are thought be more successful as compared with previous longitudinal studies in which transmastoid SS resurfacing was conducted using soft materials, namely the temporalis fascia and bone pate, reporting a success rate of 70–80% with regard to PT improvement following surgery^23^. The higher success rate observed in this study may, therefore, be due to our etiology-specific approach and secure reconstruction using firm material such as bone cement. Transmastoid SS resurfacing alone may not be sufficient to resolve PT in subjects with SS-deh, given the possible underlying mechanism of severely asymmetric or dominant flow inducing turbulent flow in the SS venous tract^5,16^. This turbulent flow may then render the SS bony wall thinner, resulting in a potential recurrence of the symptoms^19^. Therefore, transmastoid SS reshaping involving an external compression of the SS with an autologous bone chip to diminish the venous blood flow in the dominant SS may enhance treatment success rates^5^. Further, a stable and firm material, such as bone cement, may be necessary for a sustained long-term treatment effect, probably due to the risk of graft displacement or breakdown following SS resurfacing/reshaping. Indeed, bone cement has been shown to exert enhanced mechanical properties and structural integrity, reducing the chance of displacement^19^. Moreover, it is relatively easy to handle in a limited surgical field. Fueled by a correlation between material density and resurfacing outcome^20^, the firm and secure characters of bone cement may contribute to long-term PT improvement, as evidenced in this study. Nonetheless, several studies suggest that maintaining the normal diameter of the SS during surgery is imperative^25,27^. Thus, this external compression of the SS using autologous bone chips and/or bone cement should be preceded by a thorough preoperative evaluation of the vascular anatomy to assess contralateral outflow and collateral flow^28^. We believe that this precaution ensures the safety of the procedure and avoids severe postoperative complications such as increased intracranial pressure and sinus thrombosis.

### Clinical implications of objective tests for PT

Although transmastoid SS resurfacing/reshaping demonstrates favorable outcomes in most studies, not all patients with PT and SS-Div/SS-Deh experience complete resolution of symptoms after this surgery^23,25^. In addition, a discrepancy between the radiological findings and symptoms has been reportedly observed in nearly half of the patients^29^, necessitating objective tests in order to identify whether vascular pathologies on radiological modalities are causative lesions for the generation of PT, and to verify if surgical interventions really improve the symptom. For example, Subject 1 suffered from right-sided PT, and was diagnosed with SS-Div/SS-Deh and HJBD simultaneously. After transmastoid SS resurfacing/reshaping surgery, her symptom remained largely unchanged. However, it abated following transtympanic jugular bulb resurfacing. Therefore, objective tests such as TSR-STA may enable us to predict the origins at or near the middle ear and conduct surgery according to the results of the tests. Furthermore, the documentation of changes in PT symptoms using questionnaires alone is limited because of the inherent subjective nature of the questionnaires and the potentially different hemodynamic mechanisms by which each vascular abnormality may elicit PT. In the current study, TSR-STA was performed for pre and postoperative assessments in seven patients. By analyzing these results, we observed that peak and RMS amplitudes decreased significantly after transmastoid SS resurfacing/reshaping surgeries, which is in line with the subjective improvement in PT. Furthermore, the average post-treatment pure-tone threshold at 250 Hz showed significant improvement compared to the pretreatment threshold, designating the normalization of the pseudo-low frequency hearing loss due to the masking effects of the pulsatile sounds. These findings suggest that changes in peak/RMS amplitudes as well as low-frequency thresholds may be applicable in the objective diagnosis and evaluation of the effects of the treatment. Therefore, the objective measurement of PT by TSR-STA or audiometry may be of help in selecting appropriate surgical candidates, and in the objective evaluation of the treatment outcome.

## Limitations and future perspectives

There are several potential limitations in this study that should be addressed in the future. First, we observed that the improvement of PT was more appreciable at the long-term follow-up than at the short-term follow-up. Interestingly, the propensity of gradual PT improvement, if not all, was also corroborated with a recent study by Zhang et al.^30^. Although it remains elusive, this may be related to transient fluid collection in the middle ear after mastoidectomy or transient increased intracranial hypertension due to routine bandage compression after mastoidectomy, precluding complete resolution of PT symptoms. In such cases, a recent hypothetical study would offer possible explanations in which variation of SS wall pressure difference between preoperatively and postoperatively or disordered blood flow in SS might link to the discrepancy in therapeutic effects after reshaping or resurfacing surgery^31^. Also, recent resting-state functional magnetic resonance imaging (fMRI) studies have demonstrated neuropathological processes and neural network modifications in patients suffering from PT^32,33^, suggesting that the gradual improvement after surgery may be partly attributable to the reversal of the plastic changes within the brain by reducing the peripheral sound source surgically. To verify this assumption, future studies involving thorough pre and postoperative middle ear status examinations and repeated fMRIs are warranted. Second, we analyzed independent demographic and clinical factors that may have affected surgical outcome, but failed to reveal any statistically significant contributors. A lack of comprehensive demographic information and a relatively small cohort size may have limited our results, due to the retrospective nature of the study. Therefore, a prospective study comprising a large cohort is required. Furthermore, 7 of the 20 patients in this study were not evaluated by preoperative brain MRI/MRA or cerebral angiography because TB-CTA indicated definite SS-Div/SS-Deh that seemed to be pathognomonic with regard to the generation of PT. In future follow-up studies, all patients who undergo transmastoid SS resurfacing/reshaping with bone cement should be evaluated by a meticulous preoperative vascular study to review contralateral outflow and collateral flow. This comprehensive preoperative evaluation comprising cerebral angiography, or at least MRA, will ensure the safety of the procedure by detecting the possible coexistence of combined arteriovenous fistula or TS/SS stenosis^6^.

## Conclusion

In accordance with our previous preliminary reports, the long-term follow-up data obtained from this study further confirms that the surgical management of PT originating from SS-Div/SS-Deh is successful with regard to both objective and subjective measures. Also, objective measures such as pre and postoperative comparisons of TSR-STA and audiometric evaluation have been found to be effective in clarifying the effectiveness of surgical treatment in subjects with SS-Div/SS-Deh. Follow-up studies in a larger number of cases are required to further confirm the current findings.

## Methods

### Participants

We retrospectively reviewed 36 PT patients who were diagnosed with unilateral SS-Div/SS-Deh, and underwent transmastoid SS resurfacing/reshaping using bone cement between September 2014 and April 2020. Of them, 20 patients who were followed up for more than 1 year after surgery were included in the current study. The study protocol and a waiver of consent for this retrospective chart review were approved by the review board of the Clinical Research Institute at Seoul National Bundang Hospital (approval no. IRB-B-2005/613-105). All methods employed in this study were in accordance with the approved guidelines and the Declaration of Helsinki.

### Diagnosis and radiological analysis

SS-Div/SS-Deh in all patients was diagnosed by contrast-enhanced computed tomographic angiography (TB-CTA) of the temporal bone using Philips 128 CT scanners (Philips Medical Systems). TB-CTA was carried out with a tube energy of 120 kVp, a quality reference value of 250 mA, and a detector configuration of 2 × 0.625 mm. The interval of each CT scan was 0.7 mm and the length of each scan was 16 cm. We defined SS-Deh by direct contact of the SS with mastoid air cells, focal thinning of the mastoid cortex, and the absence of hyperdense bony septa overlying the SS by at least two consecutive 0.7 mm cuts on the axial TB-CTA^5^. SS-Div was defined by a focal outpouching of the SS that protruded into the mastoid air cells, resulting in the loss of the overlying calvarial cortex. Furthermore, the coexistence of TSS and an empty sella in SS-Div/SS-Deh was identified blindly using brain MRI/MRA.

### Surgical intervention

During the transmastoid SS resurfacing for patients with SS-Div, the SS and its diverticulum were skeletonized while performing cortical mastoidectomy. After complete skeletonization, the diverticulum was manually reduced down to the level of the adjacent SS wall, and the bony defect around the diverticulum was then reconstructed via the extraluminal placement of a piece of temporalis fascia and Mimix hydroxyapatite (HA) bone cement (W. Lorenz Surgical, Jacksonville, FL) (Fig. 1a). Meanwhile, during the transmastoid SS reshaping for patients with SS-Deh, the SS and its dehiscent or thinned portions were also completely skeletonized while performing simple mastoidectomy. After gentle decompression of the dominant SS by inserting a piece of harvested autologous cortical bone chip between the SS vessel wall and the thinned bony shell of the SS, reconstruction of the dehiscent or thinned bony wall was carried out using a piece of temporalis fascia and Mimix HA bone cement (Fig. 1b). The surgical procedures have previously been described in literature^5,15^.

### Evaluation of subjective symptoms

In this study, after SS resurfacing/reshaping, short-term (< 1 week) and long-term (> 1 year) changes in subjective symptoms were analyzed in all patients. The severity of PT was assessed subjectively by qualifying the loudness and annoyance of tinnitus via VAS ranging from 0 to 10^34,35^. All questionnaires were delivered the day before the operation, and during short-term (< 1 week) and long-term (> 1 year) follow-ups. Long-term postoperative outcomes were determined using VAS loudness at the last follow-up (> 1 year), and were classified into four groups: cured (100% resolution of PT), much improved (50%-100% resolution of PT), slightly improved (0%-50% resolution of PT) and stationary (no change or aggravation)^6,36^.

### Evaluation of objective parameters

At the initial visit, all patients underwent physical examination to identify any objective signs of PT (comprising head rotation to the ipsilateral and contralateral sides, digital compression of the ipsilateral internal jugular vein, and an otoendoscopic examination). All enrolled patients underwent audiometric evaluations including PTA, according to our reported protocols^37–40^. Pseudo-low frequency hearing loss (pseudo-LFHL) (an ipsilateral hearing threshold greater than 10 dB HL at both 250 and 500 Hz, or greater than 20 dB HL at either 250 or 500 Hz compared with the contralateral side) in the ipsilesional ear and its improvement after surgery were evaluated according to our previous report^17^.

TSR-STA was performed to objectively confirm PT and to analyze the psychoacoustic characteristics of the PT, following published protocols^5,18^. An external auditory canal (EAC)-sealing omnidirectional condenser microphone was used to record the sound pressure waves generated by the PT. A lavalier microphone (RODE Microphones, Sydney, Australia) was used to record the sounds. The signals were recorded using Cubase 5.0 software (Steinberg Media Technologies GmbH; Hamburg, Germany) at a sampling rate of 44,100 Hz, and the recorded signals were analyzed using MATLAB R2013a software (MathWorks, Natick, MA). Further details of TSR-STA are available in previous literature^2,6,18^.

### Statistical analysis

All data are presented as means ± standard deviation. All statistical analyses were performed using R Statistical Software (R version 3.5.2: Foundation for Statistical Computing, Vienna, Austria) and RStudio (RStudio-1.2.5042: Integrated Development for R. RStudio, Inc., Boston, MA URL https://www.rstudio.com/). A Wilcoxon-signed rank test (two-tailed) was used to evaluate changes in VAS scores between preoperative values and the short-term and long-term follow-up values. We compared the demographic data and clinical characteristics of the cured group and the much or less improved groups using an independent t-test, Mann–Whitney U test, and Chi-square test, as appropriate. Moreover, multiple logistic regression analysis was performed to simultaneously assess the relative influence of each variable. In addition, the Wilcoxon signed-rank sum test was used to evaluate differences in low frequency (250 Hz) hearing threshold between pre and post-treatment PTA, and differences in peak and RMS amplitudes between pre and post-treatment signals. *P* values < 0.05 were considered to indicate statistical significance.

## Supplementary information

Supplementary Information 1.

Supplementary Information 2.

## Data Availability

The datasets generated during and/or analyzed during the current study are available from the corresponding author on reasonable request.
