# Supplementary material for: Longitudinal analysis of surgical outcome in subjects with pulsatile tinnitus originating from the sigmoid sinus
Source: Sci Rep. 2020 Oct 23;10:18194. doi: 10.1038/s41598-020-75348-3 (PMC7584625; doi:10.1038/s41598-020-75348-3)
Supplement: Supplementary file 1 — Supplementary Information 1. [file 41598_2020_75348_MOESM1_ESM.pdf]

Longitudinal analysis of surgical outcome in subjects with pulsatile tinnitus originating from the sigmoid sinus

Sang-Yeon Lee[1], Min-Kyung Kim[1], Yun Jung Bae[2], Gwang Seok An[3], Kyogu Lee[3], Byung Yoon Choi[1],

Ja-Won Koo[1], and Jae-Jin Song[1]*

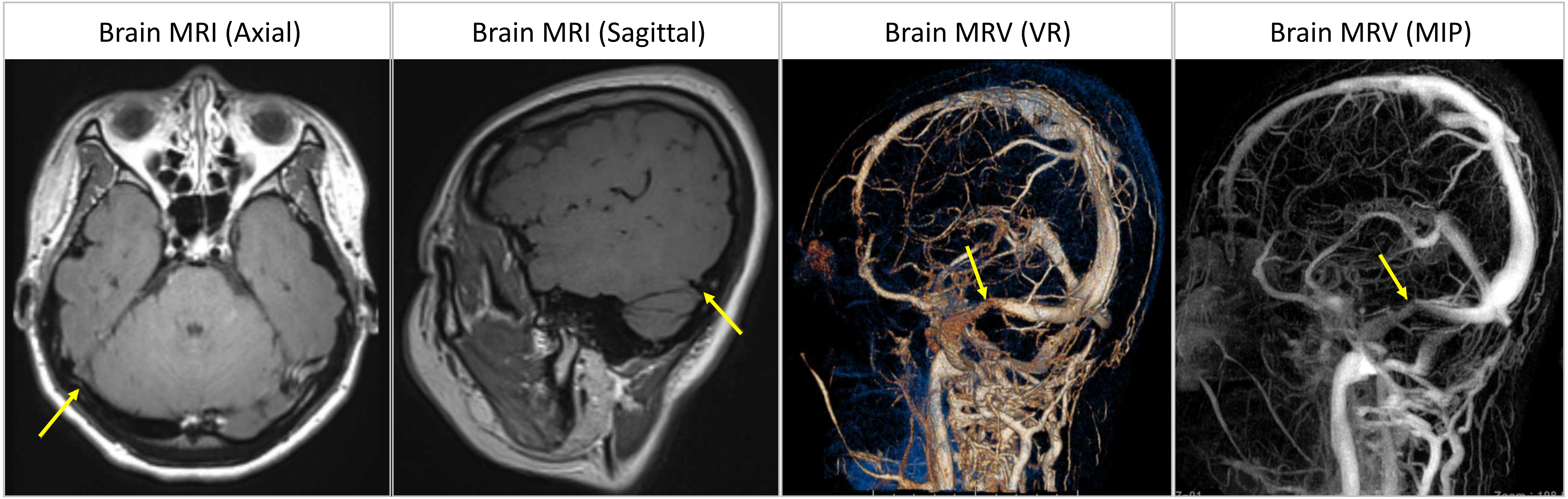

Figure S1. Representative images of transverse sinus stenosis in a subject with pulsatile tinnitus originating from a dominant sigmoid sinus with bony dehiscence. Corresponding yellow arrows on various reformations indicate transverse sinus stenosis. MRI, magnetic resonance imaging; VR, virtual reality, MIP, maximal intensity projection
