# Supplementary material for: Longitudinal analysis of surgical outcome in subjects with pulsatile tinnitus originating from the sigmoid sinus
Source: Sci Rep. 2020 Oct 23;10:18194. doi: 10.1038/s41598-020-75348-3 (PMC7584625; doi:10.1038/s41598-020-75348-3)
Supplement: Supplementary file 2 — Supplementary Information 2. [file 41598_2020_75348_MOESM2_ESM.pdf]

Longitudinal analysis of surgical outcome in subjects with pulsatile tinnitus originating from the sigmoid sinus

Sang-Yeon Lee[1], Min-Kyung Kim[1], Yun Jung Bae[2], Gwang Seok An[3], Kyogu Lee[3], Byung Yoon Choi[1],

Ja-Won Koo[1], and Jae-Jin Song[1]*

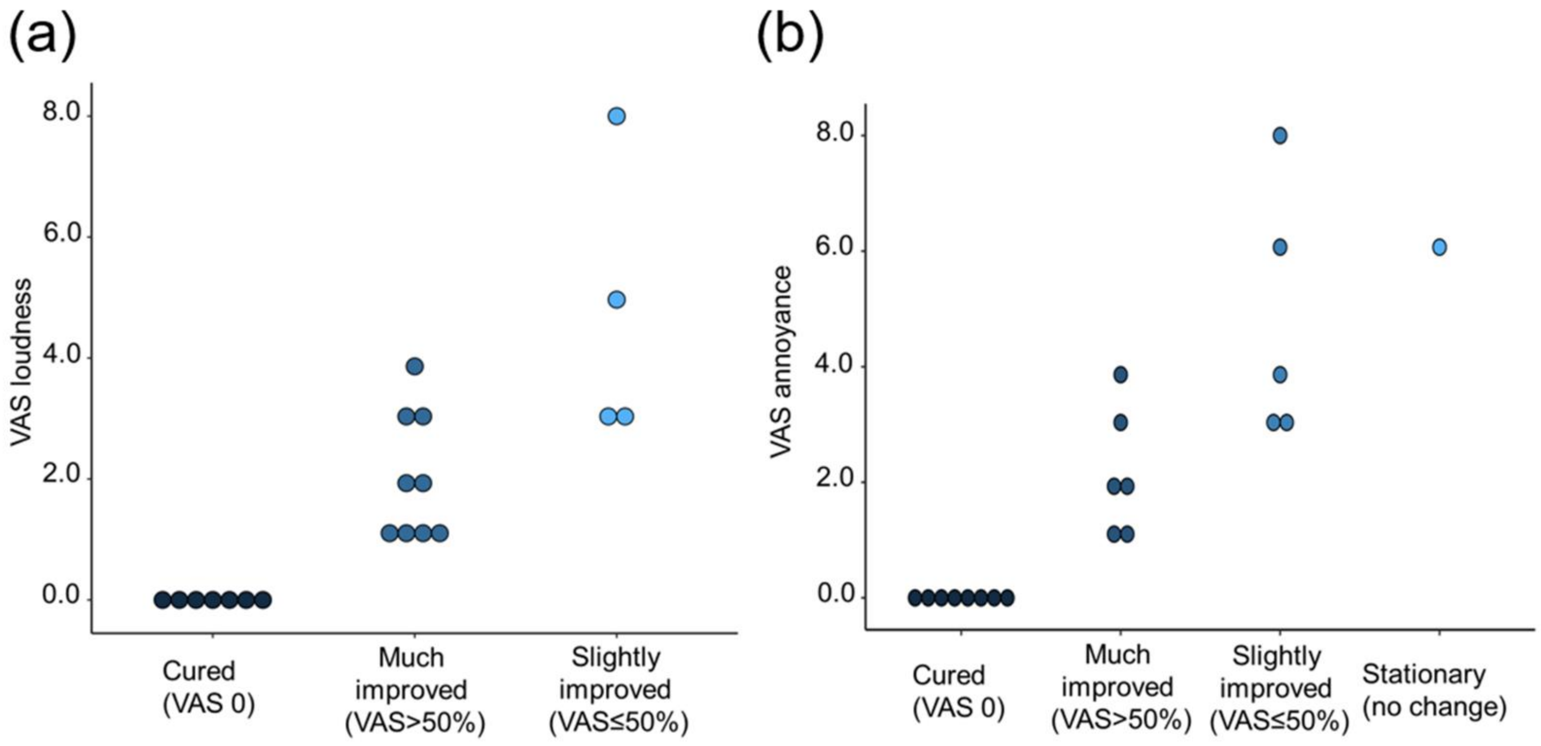

Figure S2. Sub-classification of the long-term postoperative outcomes (>1 year) based on Visual Analog Scale (VAS) loudness: “cured” (100% resolution of pulsatile tinnitus (PT)), “much improved” (50%-100% resolution of PT), “slightly improved” (0%-50% resolution of PT) and “stationary” (no change or aggravation).
